# Importance of modelling decisions on estimating trajectories of depressive symptoms and co-morbid conditions in older adults: Longitudinal studies from ten European countries

**DOI:** 10.1371/journal.pone.0214438

**Published:** 2019-04-03

**Authors:** Alejandra Marroig, Iva Čukić, Annie Robitaille, Andrea Piccinin, Graciela Muniz Terrera

**Affiliations:** 1 Instituto de Economia, Universidad de la Republica del Uruguay, Montevideo, Uruguay; 2 Centre for Dementia Prevention, University of Edinburgh, Edinburgh, United Kingdom; 3 Department of Psychology, University of Victoria, British Columbia, Canada; National Center for Child Health and Development, JAPAN

## Abstract

**Background:**

International comparisons of trajectories of depressive symptoms in older adults are scarce and longitudinal associations with co-morbid conditions not fully understood.

**Objective:**

To compare trajectories of depressive symptoms from participants living in 10 European Countries and identify ages at which the associations of co-morbid conditions with these trajectories become more relevant.

**Methods:**

Latent growth curve models were fitted to depressive symptoms scores from participants of the Survey of Health and Retirement in Europe (SHARE) initiative (combined *n* = 21,253) and co-morbid conditions modelled as time varying covariates. To identify the ages at which the association between co-morbid conditions and depressive symptoms was significant the Johnson-Neyman (JN) technique was used.

**Results:**

The shape of depressive symptoms trajectories varied between countries, and was highly dependent on modelling decisions. The association between the average number of co-morbidities reported over time and depressive symptoms was consistent and positive across countries and ages.

**Conclusion:**

International differences in ageing-related trajectories of depressive symptoms emerged. The longitudinal association of co-morbid conditions with trajectories of depressive symptoms was found, but the results overall suggest that modelling decisions could greatly influence the outcomes, and should thus be interpreted with caution.

## Introduction

Depression, a mood disorder that causes a persistent feeling of sadness and loss of interest in activities, is a major contributor to the burden of disease world wide [1,2]. In older adults, several chronic conditions including diabetes, ischaemic heart disease, heart failure, chronic obstructive pulmonary, Parkinson’s disease, stroke have been identified as independent risk factors for depression [3]. However, in middle- and older aged adults, these conditions tend to coexist. The presence of several chronic conditions has been shown to increase the risk of depression over and above having a single one of the conditions [4] and the prevalence of multiple co-morbid conditions increases with age [5] making the association between the presence of multiple conditions and depression in older adults particularly relevant. This further escalates as the number of depressive symptoms reported by older adults grows in an accelerating manner with increasing age [6] and individuals report a varying number of co-morbid conditions over time [7]. Therefore, if the association between depressive symptoms and co-morbid conditions is only looked at a single point in time, the dynamics of the association between depressive symptoms and multimorbidity is overlooked. Hence, to fully understand the association between the presence of multiple chronic conditions and depressive symptomatology in older adults it becomes essential to evaluate longitudinal measurements of both, and apply adequate statistical methodologies that account for their changing nature.

Rast et al [8] examined changes in depressive symptoms as individuals approach death in a sample of deceased participants of the Health and Retirement Study (HRS). They fitted a linear mixed model to depressive symptoms scores expressed as a function of years to death and treated co-morbidities as time varying covariates. This modeling approach allows understanding the change in depressive symptoms as individuals approach death, and obtaining separate estimates of how the effect of co-morbid conditions on depressive symptoms varies over time. To examine whether the change in depressive symptoms scores may be better described as a non-linear, they modelled their trajectory with a second order polynomial.

The use of second order polynomials for modelling nonlinear trajectories is common in research. In a second order polynomial, the zero order term (intercept) represents the level of the outcome (conditional on covariates) at time 0, the first order term (linear slope) represents the instantaneous rate of change at time 0 and the second order term (quadratic slope) represents a factor of the change in rate of change (acceleration or deceleration) over time. By definition, the linear slope in a second order polynomial is dependent on the placement of the intercept and its statistical significance may also be dependent on such placement. That is, it is possible that for some values of the intercept, the linear slope does not reach statistical significance whereas it does reach significance levels for other values of the intercept. Even more, the association of covariates with the linear slope may also be dependent on the placement of the intercept. Although regions of significance of the linear slope could be identified using a pick a point approach [9], where the intercept is placed at different values and models re-estimated for each of these values, the JN technique [10] facilitates their estimation. This simple technique permits the calculation of regions of significance for a simple slope of a focal predictor on an outcome variable across a range of the second continuous independent variable. Rast et al. used this technique to “identify conditions and times under which multimorbidity plays a role in depression… and when such an association may require less attention or less treatment” [8].

Although Rast and colleagues’ work provides important insights into the interplay between changes in depressive symptoms and multimorbidities, it does so in the context of changes before death and uses data from deceased individuals from a single study. In the current study, we aim to examine trajectories of change in depressive symptoms whilst accounting for the number of multimorbidities individuals report. In addition, we aim to identify ages at which the association between multimorbidities and depressive symptoms may require more or less attention in the context of ageing-related changes in depressive symptoms. Finally, we were able to replicate our models using data from ten European countries that participate in the Survey of Health, Ageing and Retirement in Europe (SHARE, www.share-project.org). Importantly, the use of such harmonized dataset maximised our ability to examine replicability of results, across modelling decisions and different socio-cultural backgrounds.

## Method

### Participants

The participants were drawn from the SHARE consortia, where information on health, socio-economic status and social and family networks of more than 85,000 individuals aged 50 and over has been collected since 2004 across 20 European countries. The ethical approval was granted by the Ethics Committee of the University of Mannheim for Waves 1–4. Wave 4 and the continuation of the project were further approved by the Ethics Council of the Max Plank Society. For each participating country, a separate approval was obtained by the respective ethics committees whenever it was required (for more details on the ethical approvals see: http://www.share-project.org/fileadmin/pdf_documentation/SHARE_ethics_approvals.pdf).

In the current study, we included data from 10 countries (Austria, Belgium, Denmark, France, Germany, Italy, Netherlands, Spain, Sweden and Switzerland) where information about depressive symptoms was collected in 4 occasions (waves 1, 2, 4 and 5). The total analytic sample from all ten countries combined is *n* = 21,253.

*Depressive symptoms*: The EURO-D symptom scale measures the current depression and is constructed as a composite of 12 items: depressed mood, pessimism, suicidality, guilt, sleep, interest, irritability, appetite, fatigue, concentration, enjoyment and tearfulness. It takes values from 0 (not depressed) to 12 (very depressed).

*Multimorbidities*: At each data wave, participants were asked if the doctor ever told them that they had: a heart attack, high blood pressure or hypertension, high cholesterol, a stroke or cerebral vascular disease, diabetes or high blood sugar, chronic lung disease, cancer or malignant tumour, stomach or duodenal ulcer, peptic ulcer, Parkinson disease, cataracts, hip fracture or femoral fracture.

Table 1 shows the mean and standard deviation of depressive symptoms and chronic diseases at study entry and individual sample sizes across all ten countries.

**Table 1:** Depressive symptoms (EURO-D) over time by country.

	Wave 1			Wave 2			Wave 3			Wave 4		
	Mean(st.dev)			Mean(st.dev)			Mean(st.dev)			Mean(st.dev)		
	Depressive symptoms	Chronic conditions	N	Depressive symptoms	Chronic Conditions	N	Depressive symptoms	Chronic conditions	N	Depressive symptoms	Chronic conditions	N
Austria	2.29(2.13)	0.99(1.07)	1354	2.22(2.16)	1.07(1.12)	1056	2.45(2.03)	1.24(1.26)	4146	2.26(2.02)	1.22(1.23)	3337
Belgium	2.53(2.12)	1.17(1.17)	3306	2.54(2.22)	1.14(1.15)	2810	2.83(2.20)	1.26(1.25)	4614	2.81(2.22)	1.19(1.20)	4801
Denmark	2.07(1.92)	1.07(1.22)	1381	2.06(1.84)	1.07(1.23)	2135	1.99(1.86)	1.01(1.16)	1864	2.23(1.86)	1.16(1.26)	3066
France	3.00(2.25)	1.05(1.13)	2614	2.86(2.29)	1.01(1.08)	2499	3.09(2.27)	1.17(1.20)	4934	2.98(2.24)	1.10(1.15)	3964
Germany	2.30(1.99)	1.11(1.17)	2378	2.20(1.92)	1.08(1.15)	2145	2.46(1.99)	1.32(1.26)	1411	2.81(1.94)	1.33(1.30)	4486
Italy	3.09(2.45)	1.12(1.18)	2276	2.97(2.53)	1.18(1.23)	2643	2.94(2.41)	1.17(1.18)	3118	3.34(2.52)	1.19(1.22)	3961
Netherlands	2.26(1.99)	0.96(1.12)	2388	2.12(1.94)	0.87(1.08)	2242	2.10(1.92)	0.98(1.14)	2352	2.24(1.93)	1.03(1.19)	3282
Spain	3.41(2.71)	1.23(1.24)	2090	2.98(2.63)	1.08(1.14)	1950	3.16(2.65)	1.36(1.29)	2990	3.10(2.59)	1.32(1.26)	4878
Sweden	2.20(1.90)	1.06(1.19)	2647	2.02(1.83)	1.08(1.20)	2365	2.15(1.86)	1.13(1.22)	1771	2.37(1.81)	1.19(1.27)	3641
Switzerland	2.12(1.80)	0.78(0.99)	819	1.99(1.86)	0.76(0.96)	1245	2.27(1.82)	0.88(1.06)	3124	2.09(1.77)	0.79(1.00)	2581

### Statistical analysis

Independent linear mixed models were fitted to depressive symptoms scores from each country as a function of age at each occasion. The equation for each country is specified as follows:

$$Y_{ij}=\beta_{0}+\beta_{1}{Age}_{ij}+\beta_{2}{Age}_{ij}^{2}+\beta_{3}{MMa}_{i}+\beta_{4}{MMc}_{ij}+\beta_{5}{BA}_{i}+\beta_{6}{Age}_{ij}\times {MMa}_{i}+\beta_{7}{Age}_{ij}\times {MMc}_{ij}+\beta_{8}{Age}_{ij}\times {BA}_{i}+\beta_{9}{Age}_{ij}^{2}\times {MMa}_{i}+\beta_{10}{Age}_{ij}^{2}\times {MMc}_{ij}+\beta_{11}{Age}_{ij}^{2}\times {BA}_{i}+\beta_{12}{MMa}_{i}\times {BA}_{i}+\beta_{13}{MMc}_{ij}\times {BA}_{i}+\beta_{14}{Age}_{ij}\times {MMa}_{i}\times {BA}_{i}+\beta_{15}{Age}_{ij}\times {MMc}_{ij}\times {BA}_{i}+b_{0i}+b_{1i}{Age}_{ij}+\varepsilon_{ij}$$

where Y_ij_ represents the value of EURO-D scale for individual i in occation j, b_0i_ and b_1i_ are random effects for intercept and linear slope of age and ε_ij_ a normal error term. To account for age differences at study entry, we adjusted the level and linear and quadratic slopes for baseline age (BA). Following Rast’s model, we adjusted curve parameters for a time invariant variable representing the average number of multimorbidities per person across time (MMa). We also included a time varying variable (MMc) that represents occasion-specific deviations in multimorbidity from a person’s average number of multimorbidities over time and added second and third order interaction terms with baseline age and MMa. Data from individuals who did not report multimorbidities were excluded from the analysed sample.

We set the model intercept at three different values 5 years apart: 60, 65 and 75 years old and also applied the JN technique. The placement of the intercept at these different ages and the application of the JN technique allowed us to examine the significance of the linear slope at a whole range of ages.

All analyses were conducted in Stata 13 (StataCorp, 2013) using the command xtmixed. Models were estimated using maximum likelihood estimation, under the assumption of missing at random missing data.

## Results

The level of depressive symptoms and number of co-morbid conditions at study entry varied across countries (*p* < 0.05). At study entry and over the entire follow up period, Spain, Italy and France were the countries with the highest average number of depressive symptoms, and Denmark the country with the lowest average number of depressive symptoms. Also consistently over the entire follow up period, Spain was the country with the highest average number of chronic conditions and Switzerland the country with the lowest average number of chronic conditions (Table 1).

*Trajectories of depressive symptoms*. Trajectories of depressive symptoms were inconsistent across countries. Within countries, they also depended on the intercept placement. (See Tables 2 and 3).

**Table 2:** Fixed effects estimates for EURO-D.

	Austria Intercept placed at			Belgium Intercept placed at			Denmark Intercept placed at			France Intercept placed at			Germany Intercept placed at		
Fixed effects	Age 60	Age 65	Age 70	Age 60	Age 65	Age 70	Age 60	Age 65	Age 70	Age 60	Age 65	Age 70	Age 60	Age 65	Age 70
Age	-0.04**	-0.01	0.01	-0.03**	-0.01	0.02**	-0.04***	-0.02**	-0.01	-0.01	0.00	0.02**	-0.01	0.01	0.03***
	(0.02)	(0.01)	(0.01)	(0.01)	(0.01)	(0.01)	(0.01)	(0.01)	(0.01)	(0.01)	(0.01)	(0.01)	(0.01)	(0.01)	(0.01)
Baseline Age	0.02	0.02*	0.03**	0.00	0.00	-0.01	0.01	0.01	0.01	-0.01	-0.01	-0.01	-0.02	-0.01	-0.01
	(0.02)	(0.01)	(0.01)	(0.01)	(0.01)	(0.01)	(0.01)	(0.01)	(0.01)	(0.01)	(0.01)	(0.01)	(0.01)	(0.01)	(0.01)
MMc	0.26***	0.21***	0.19***	0.18***	0.14***	0.12***	0.18***	0.15**	0.14**	0.15**	0.14**	0.13**	0.44***	0.34***	0.26***
	(0.05)	(0.05)	(0.05)	(0.04)	(0.04)	(0.04)	(0.06)	(0.06)	(0.06)	(0.05)	(0.05)	(0.05)	(0.06)	(0.06)	(0.06
MMa	0.37***	0.34***	0.32***	0.48***	0.43***	0.40***	0.35***	0.29***	0.26***	0.40***	0.37***	0.34***	0.38***	0.34***	0.30***
	(0.03)	(0.03)	(0.03)	(0.03)	(0.03)	(0.03)	(0.03)	(0.03)	(0.03)	(0.03)	(0.03)	(0.04)	(0.03)	(0.03)	(0.03)
Age2	0.00**	0.00*	0.00	0.00***	0.00***	0.00***	0.00*	0.00*	0.00	0.00*	0.00**	0.00*	0.00	0.00	0.00
	(0.00)	(0.00)	(0.00)	(0.00)	(0.00)	(0.00)	(0.00)	(0.00)	(0.00)	(0.00)	(0.00)	(0.00)	(0.00)	(0.00)	(0.00)
Age x Baseline Age	0.00	0.00	0.00	0.00	0.00	0.00	0.00	0.00	0.00	0.00	0.00	0.00	0.00	0.00	0.00
	(0.00)	(0.00)	(0.00)	(0.00)	(0.00)	(0.00)	(0.00)	(0.00)	(0.00)	(0.00)	(0.00)	(0.00)	(0.00)	(0.00)	(0.00)
Age x MMc	0.00	0.00	0.01	-0.02	-0.01	0.00	-0.01	-0.01	-0.02	0.01	0.01	0.01	-0.06**	-0.05***	-0.04***
	(0.02)	(0.02)	(0.01)	(0.01)	(0.01)	(0.01)	(0.02)	(0.01)	(0.01)	(0.02)	(0.01)	(0.01)	(0.02)	(0.02)	(0.01)
Age x MMa	-0.01	0.00	0.01	-0.01	-0.01	-0.01	-0.01	-0.01	0.00	-0.02	-0.01	0.00	0.00	0.00	0.00
	(0.01)	(0.01)	(0.01)	(0.01)	(0.01)	(0.01)	(0.01)	(0.01)	(0.01)	(0.01)	(0.01)	(0.01)	(0.01)	(0.01)	(0.01)
Baseline Age x MMc	-0.01	-0.01	-0.01	0.00	0.00	0.00	0.00	0.01	0.01	-0.02	-0.01	-0.01	0.04*	0.03**	0.02*
	(0.02)	(0.02)	(0.01)	(0.01)	(0.01)	(0.01)	(0.02)	(0.01)	(0.01)	(0.02)	(0.01)	(0.01)	(0.02)	(0.01)	(0.01)
Baseline Age x MMa	0.00	-0.01	-0.01	0.00	0.00	0.00	0.00	0.00	-0.01	0.01	0.00	0.00	-0.01	-0.01	-0.01
	(0.01)	(0.01)	(0.01)	(0.01)	(0.01)	(0.01)	(0.01)	(0.01)	(0.01)	(0.01)	(0.01)	(0.01)	(0.01)	(0.01)	(0.01)
Baseline Age x Age2	0.00**	0.00**	0.00**	0.00	0.00	0.00	0.00	0.00	0.00	0.00	0.00	0.00	0.00	0.00	0.00
	(0.00)	(0.00)	(0.00)	(0.00)	(0.00)	(0.00)	(0.00)	(0.00)	(0.00)	(0.00)	(0.00)	(0.00)	(0.00)	(0.00)	(0.00)
MMc x Age2	0.00	0.00	0.00	0.00	0.00	0.00	0.00	0.00	0.00	0.00	0.00	0.00	0.00	0.00	0.00
	(0.00)	(0.00)	(0.00)	(0.00)	(0.00)	(0.00)	(0.00)	(0.00)	(0.00)	(0.00)	(0.00)	(0.00)	(0.00)	(0.00)	(0.00)
Mma x Age2	0.00	0.00	0.00	0.00	0.00	0.00	0.00	0.00	0.00*	0.00**	0.00**	0.00**	0.00	0.00	0.00
	(0.00)	(0.00)	(0.00)	(0.00)	(0.00)	(0.00)	(0.00)	(0.00)	(0.00)	(0.00)	(0.00)	(0.00)	(0.00)	(0.00)	(0.00)
Age x Baseline Age x MMa	0.00	0.00	0.00	0.00	0.00	0.00	0.00	0.00	0.00	0.00	0.00	0.00	0.00	0.00	0.00
	(0.00)	(0.00)	(0.00)	(0.00)	(0.00)	(0.00)	(0.00)	(0.00)	(0.00)	(0.00)	(0.00)	(0.00)	(0.00)	(0.00)	(0.00)
Age x Baseline Age x MMc	0.00	0.00	0.00	0.00	0.00	0.00	0.00	0.00	0.00	0.00	0.00	0.00	0.00	0.00	0.00
	(0.00)	(0.00)	(0.00)	(0.00)	(0.00)	(0.00)	(0.00)	(0.00)	(0.00)	(0.00)	(0.00)	(0.00)	(0.00)	(0.00)	(0.00)
Intercept	1.79***	1.77***	1.90***	2.18***	2.09***	2.09***	1.80***	1.68***	1.64***	2.49***	2.43***	2.46***	2.06***	1.98***	2.01***
	(0.05)	(0.05)	(0.06)	(0.05)	(0.05)	(0.06)	(0.05)	(0.06)	(0.06)	(0.05)	(0.06)	(0.06)	(0.05)	(0.05)	(0.05)

Note. Standard errors in parentheses.

* p< 0.10

** p < 0.05

*** p < 0.01

**Table 3:** Fixed effects estimates for EURO-D.

	Italy Intercept placed at:			Netherlands Intercept placed at:			Spain Intercept placed at:			Sweden Intercept placed at:			Switzerland Intercept placed at:		
Fixed effects	Age 60	Age 65	Age 70	Age 60	Age 65	Age 70	Age 60	Age 65	Age 70	Age 60	Age 65	Age 70	Age 60	Age 65	Age 70
Age	0.00	0.01	0.02**	-0.05***	-0.04***	-0.03***	-0.08***	-0.06***	-0.04***	-0.05***	-0.03***	0.00	-0.04***	-0.02*	0.00
	(0.01)	(0.01)	(0.01)	(0.01)	(0.01)	(0.01)	(0.02)	(0.01)	(0.01)	(0.01)	(0.01)	(0.01)	(0.01)	(0.01)	(0.01)
Baseline Age	-0.01	0.00	0.02**	0.04***	0.04***	0.04***	0.08***	0.08***	0.08***	0.02*	0.02**	0.01*	0.02	0.02	0.01
	(0.02)	(0.01)	(0.01)	(0.01)	(0.01)	(0.01)	(0.02)	(0.01)	(0.01)	(0.01)	(0.01)	(0.01)	(0.01)	(0.01)	(0.01)
MMc	0.35***	0.26***	0.20***	0.11*	0.12**	0.12**	0.49***	0.49***	0.48***	0.17***	0.16***	0.14**	0.25***	0.21***	0.16**
	(0.06)	(0.06)	(0.06)	(0.05)	(0.05)	(0.05)	(0.07)	(0.06)	(0.06)	(0.06)	(0.05)	(0.05)	(0.07)	(0.06)	(0.06)
MMa	0.57***	0.57***	0.57***	0.35***	0.33***	0.30***	0.52***	0.51***	0.50***	0.26***	0.24***	0.24***	0.39***	0.33***	0.29***
	(0.04)	(0.03)	(0.04)	(0.03)	(0.03)	(0.03)	(0.04)	(0.03)	(0.04)	(0.03)	(0.03)	(0.03)	(0.04)	(0.04)	(0.04)
Age2	0.00	0.00	0.00	0.00	0.00	0.00	0.00	0.00	0.00	0.00***	0.00***	0.00***	0.00**	0.00**	0.00*
	(0.00)	(0.00)	(0.00)	(0.00)	(0.00)	(0.00)	(0.00)	(0.00)	(0.00)	(0.00)	(0.00)	(0.00)	(0.00)	(0.00)	(0.00)
Age x Baseline Age	0.00**	0.00**	0.00***	0.00	0.00	0.00	0.00	0.00	0.00	0.00	0.00	0.00	0.00	0.00	0.00
	(0.00)	(0.00)	(0.00)	(0.00)	(0.00)	(0.00)	(0.00)	(0.00)	(0.00)	(0.00)	(0.00)	(0.00)	(0.00)	(0.00)	(0.00)
Age x MMc	-0.04**	-0.03*	-0.02	0.02	0.02	0.02	0.00	-0.01	-0.01	-0.01	0.00	0.00	0.00	0.00	0.00
	(0.02)	(0.02)	(0.01)	(0.02)	(0.01)	(0.01)	(0.02)	(0.02)	(0.01)	(0.02)	(0.01)	(0.01)	(0.03)	(0.02)	(0.02)
Age x MMa	0.01	0.01*	0.02**	0.00	0.00	0.00	0.01	0.01	0.02*	0.01	0.01	0.01	-0.04**	-0.02*	-0.01
	(0.01)	(0.01)	(0.01)	(0.01)	(0.01)	(0.01)	(0.01)	(0.01)	(0.01)	(0.01)	(0.01)	(0.01)	(0.01)	(0.01)	(0.01)
Baseline Age x MMc	0.02	0.01	0.01	-0.02	-0.02*	-0.02*	0.00	0.01	0.01	0.00	0.00	0.00	-0.01	-0.01	-0.01
	(0.02)	(0.01)	(0.01)	(0.02)	(0.01)	(0.01)	(0.02)	(0.01)	(0.01)	(0.02)	(0.01)	(0.01)	(0.02)	(0.02)	(0.01)
Baseline Age x MMa	-0.01	-0.02*	-0.02***	0.00	0.00	-0.01	-0.01	-0.01	-0.02**	-0.01	-0.01*	-0.01**	0.02	0.01	0.00
	(0.01)	(0.01)	(0.01)	(0.01)	(0.01)	(0.01)	(0.01)	(0.01)	(0.01)	(0.01)	(0.01)	(0.01)	(0.02)	(0.01)	(0.01)
Baseline Age x Age2	0.00	0.00	0.00	0.00	0.00	0.00	0.00	0.00	0.00	0.00	0.00	0.00	0.00*	0.00	0.00
	(0.00)	(0.00)	(0.00)	(0.00)	(0.00)	(0.00)	(0.00)	(0.00)	(0.00)	(0.00)	(0.00)	(0.00)	(0.00)	(0.00)	(0.00)
MMc x Age2	0.00	0.00	0.00	0.00	0.00	0.00	0.00	0.00	0.00	0.00	0.00	0.00	0.00	0.00	0.00
	(0.00)	(0.00)	(0.00)	(0.00)	(0.00)	(0.00)	(0.00)	(0.00)	(0.00)	(0.00)	(0.00)	(0.00)	(0.00)	(0.00)	(0.00)
Mma x Age2	0.00	0.00	0.00	0.00	0.00	0.00	0.00	0.00	0.00	0.00	0.00	0.00	0.00**	0.00**	0.00**
	(0.00)	(0.00)	(0.00)	(0.00)	(0.00)	(0.00)	(0.00)	(0.00)	(0.00)	(0.00)	(0.00)	(0.00)	(0.00)	(0.00)	(0.00)
Age x Baseline Age x MMa	0.00	0.00	0.00	0.00	0.00	0.00	0.00	0.00	0.00	0.00	0.00	0.00	0.00**	0.00**	0.00**
	(0.00)	(0.00)	(0.00)	(0.00)	(0.00)	(0.00)	(0.00)	(0.00)	(0.00)	(0.00)	(0.00)	(0.00)	(0.00)	(0.00)	(0.00)
Age x Baseline Age x MMc	0.00	0.00	0.00	0.00	0.00	0.00	0.00	0.00	0.00	0.00	0.00	0.00	0.00	0.00	0.00
	(0.00)	(0.00)	(0.00)	(0.00)	(0.00)	(0.00)	(0.00)	(0.00)	(0.00)	(0.00)	(0.00)	(0.00)	(0.00)	(0.00)	(0.00)
Intercept	2.24***	2.24***	2.38***	1.97***	1.91***	1.93***	2.36***	2.39***	2.51***	1.96***	1.86***	1.87***	1.86***	1.79***	1.80***
	(0.06)	(0.06)	(0.06)	(0.05)	(0.05)	(0.06)	(0.06)	(0.06)	(0.07)	(0.04)	(0.05)	(0.05)	(0.05)	(0.05)	(0.06)

Note. Standard errors in parentheses.

* p< 0.10

** p < 0.05

*** p < 0.01

When the intercept was placed at age 60, trajectories for Austria, Belgium, Denmark, Sweden and Switzerland exhibited accelerated change, whereas in the Netherlands and Spain the quadratic term did not reach conventional statistical significance suggesting that depressive symptoms changed linearly from age 60 in these two countries. In Germany and Italy, neither slope was significant, whilst in France, change in depressive symptoms was found to consistently accelerate over time.

The placement of the intercept at age 65 impacted the estimated values for the trajectory parameters and the trajectory shape in most countries. For example, in Austria, Belgium and France, depressive symptoms accelerated at constant rate, whilst in Denmark, Sweden and Switzerland, they also accelerated but not at constant rate. In the Netherlands and Spain, depressive symptoms appeared to change linearly from age 65, and in Italy and Germany, a flat trajectory was estimated.

When the intercept was placed at age 70, trajectories of depressive symptoms exhibited accelerating change only in Belgium, France, Sweden and Switzerland. In Sweden and Switzerland the linear slopes were nonsignificant, suggesting that change in depressive symptoms constantly accelerated with age and in Austria and Denmark, trajectories were flat. In Germany, Italy, the Netherlands and Spain, trajectories exhibited linear change

*Multimorbidities and depressive symptoms trajectories*. A positive association between the average number of multimorbidities across time (MMa) and the average number of depressive symptoms at ages 60, 65 and 70 was consistently found in all 10 countries. This suggests that as the number of average multimorbidities reported over time increases, the average number of depressive symptoms at these 3 time points also increases, although estimates of these associations decreased with increasing age of intercept placement. When the intercept was placed at ages 65 and 70, the association of MMa with the average number of depressive symptoms reported at these ages was age dependent only in Italy (Baseline Age _X_ MMa). At age 70, a similar association also emerged in Spain. These findings suggest that the effect of an increasing number of multimorbidities over time on the average number of depressive symptoms at those specific ages, decreases with older age at study entry.

The association between MMa and rate of change at ages 60, 65 and 70 was not consistent across countries. For example, in Switzerland, our results indicate a negative significant association between MMa and rate of change in depressive symptoms from ages 60 and 65, but at age 70, the estimate did not reach conventional significance. Furthermore, this association was found to change with older age at study entry although the estimate of the interaction terms between baseline age, MMa and rate of change was very small. In other countries (Austria, Belgium, Denmark and France) there was also some evidence of a negative association between MMa and rate of change in depressive symptoms at age 60 although estimates did not reach conventional statistical significance. Similar nonsignificant results were found in Belgium, Denmark and France with rate of change from older ages.

In Italy, instead, MMa was found to be positively associated with rate of change at ages 65 and 70 and in Spain, with rate of change at age 70. These positive estimates suggest that rate of change in depressive symptoms from these ages increase with an increasing number of multimorbidities.

In all countries and independently of the age at which the intercept was set, in occasions where the number of multimorbidities increased, the number of depressive symptoms also increased (MMc). In most countries there was no association between occasion specific deviations from the average number of multimorbidities and the linear or quadratic slopes, regardless of the intercept placement. Exceptions to these otherwise consistent null results were linear rate of change at ages 60 and 65 years in Italy and the linear rate of change from all ages in Germany.

*Johnson-Neyman technique*: In contrast with the previous analyses that permitted the investigation of the association of multimorbidities and rate of change at three pre specified ages, results from the application of the JN technique allowed us to examine changes in instantaneous rate of change for a continuum range of different ages by a varying number of multimorbidities.

**Fig 1:**
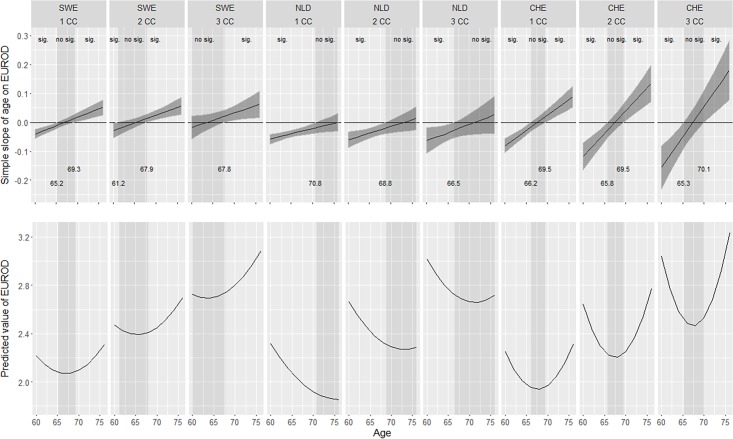
**Linear slopes of EURO-D scores for the Netherlands, Sweden and Switzerland for 1–3 chronic conditions and their corresponding confidence regions (top panel) and predicted trajectories for three different placements of the model intercept (bottom panel)**.

For the sake of brevity, we present in Fig 1 a graphical representation of results from the Netherlands, Sweden and Switzerland as these exhibited similar patters as other countries examined. Specifically, the Netherlands exhibited a pattern of change similar to Spain, Sweden to Austria and Switzerland to Belgium, Denmark and France. The top row of this picture depicts linear slopes of EURO-D scores for each of the 3 countries and for 1–3 chronic conditions from models where the intercept was placed at a range of different ages and confidence regions about these slopes (dark shaded areas). Where the confidence regions include zero, the slopes become non significantly different from zero. The ages where the linear slopes become non significantly different from zero are marked in vertical lighter-shaded bands. For instance, in the Netherlands, for individuals with 1 chronic condition in average, the linear slope of depressive symptoms becomes non significant from age 70.78 years, whilst for persons with more chronic conditions, this happens at younger ages (68.85 and 66.54 years old). The bottom panel depicts predicted trajectories for models with various intercepts and number of chronic conditions.

The bottom row of graphs depict the predicted curves for EURO-D scores for each of the 3 countries and for 1–3 chronic conditions from models where the intercept was placed at a range of different ages.

## Discussion

We evaluated the association of two dynamic processes: the number of multimorbidities individuals report with increasing age and ageing-related trajectories of depressive symptoms in 10 European countries that form part of the SHARE initiative. Using the JN technique we demonstrated the complex nature of this association and the dependency of substantive conclusions on methodological decisions.

Our results indicate international differences in depressive symptoms trajectories. Importantly, our results also demonstrate the high dependency of results on the placement of the intercept of the curve used to estimate these trajectories. For instance, whilst in Austria placing the intercept at age 60 resulted in a trajectory of depressive symptoms that exhibited some curvature and such that the linear rate of change at age 60 achieved conventional statistical significance, this linear slope became non significant when the curve’s intercept was placed at age 65 or age 70. In contrast, in Germany, depressive symptoms did not appear to change from age 60 or 65, but they declined linearly from age 70. Consistently across countries and at the ages of 60, 65 and 70 years, a positive association was found between the average number of multimorbidities over time with the average level of depressive symptoms at these same ages. These results are an illustration of the consequences of important methodological considerations that should not be overlooked when comparing results across studies. Furthermore, these illustrations also suggest the need for a thorough description of statistical analyses performed (often overlooked) when reporting results in scientific publications, so that a fair synthesis of evidence can be performed.

Our results only partially agree with Rast ‘s, who used data from the HRS (a study harmonized with SHARE) and a very similar statistical approach. Rast reported that depressive symptoms and MMc appeared to increase at the end of life whilst MMa did not appear to be associated with CES-D scores at the time of death. However, differences between their results and ours were expected as Rast and colleagues modelled change in depressive symptoms as a function of time to death, a metric of time different from age, the metric of time we used in our work. The choice of an optimal metric of time to model change is a well-studied problem [11] in the cognitive ageing literature where it has been suggested that change should be optimally modelled as a function of a metric of time that best represent the process inducing the observed changes.

When a metric of time is defined using a land marking event such as death, a health episode such as having a stroke, taking retirement, the moment time starts to be clocked (origin) defines a natural intercept. However, this is not the case of age, that although it clocks time since birth, in longitudinal studies of ageing, researchers make a somehow subjective choice about the age they start measuring the passing of time, and consequently, about the intercept placement. Such choice almost certainly impacts results as demonstrated in this paper, and this impact is also dependent on the parametric curve considered. Polynomial curves are often chosen because of ease of parameter interpretation, although when higher order polynomials are considered, the interpretation of all parameters except the highest order term is dependent on the placement of the intercept.

The JN technique we employed here allowed us to partly disentangle the complex associations between aging-related depressive symptoms and multimorbidities. For instance, our results indicate that the average number of multimorbidites an individual has over time (MMa) is consistently associated with level of depressive symptoms at ages 60, 65 and 70 years in all countries but only changed with increasing age when the intercept was placed at age 70 in Sweden, Spain and Italy such that per extra year of older age at study entry, the effect of an increasing number of multimorbidites became more severe. MMa was found to be associated with instantaneous rate of change from age 70 in Spain and Italy, and with instantaneous rate of change from age 60 in Switzerland.

### Strengths and limitations

Our work has various strengths and limitations. To begin with, a strong methodological advantage of the coordinated analytical approach considered is that results are directly comparable across countries. This is the result of the harmonized design used in SHARE, but also of the fact that as the same method was employed with covariates coded consistently across countries, estimated parameters have the same meaning across all models. This allowed for a direct pre-publication replication of the results.

Linear mixed models assume missing data are missing at random, an assumption that may be violated in longitudinal studies of older adults. Because an aim of our work was to illustrate the application of the JN technique and what can be learnt from its use, whilst aiming at evaluating whether patters of change in depressive symptoms and the impact of multimorbidity on these patterns were consistent across 10 European studies, we fitted a relatively simple model as the one employed by Rast. We encourage the further development of the models considered here in future research.

We focused on the learning gains that result from the application of the JN technique and its potential impact when synthesizing evidence. Although some of the previous points discussed may initially appear to be simple methodological considerations, we would strongly encourage researchers not to overlook them when estimating trajectories (not only of depressive symptoms as they apply to any outcome) and synthesizing evidence regarding the associations between depressive symptoms and multimorbidities, as evidence generated after fitting alternative models may not be directly comparable, and therefore, the advancement of our knowledge about these two processes may be hampered.

Finally, we are not able to test whether there is a causal link between the number of multimorbidities and depressive symptoms. While this is ultimately important as a potential target for prevention, it is important to establish what the associations look like in a population, whether they replicate in different populations and whether they are robust or dependent on experimenter degrees of freedom (i.e. modelling decisions in the current study). Establishing the true links between beteen depressive symptoms and multimorbidities is a step in the right direction towards building a causal model of the processes in question.

## References

[1] World Health Organization N: The Global Burden of Disease: 2004 update. Update 2008;2010:146.

[2] Ferrari AJ, Charlson FJ, Norman RE, Patten SB, Freedman G, Murray CJL, et al: Burden of Depressive Disorders by Country, Sex, Age, and Year: Findings from the Global Burden of Disease Study 2010. PLoS Medicine 2013;10 10.1371/journal.pmed.1001547 24223526 PMC3818162

[3] Ayuso-Mateos JL, Nuevo R, Verdes E, Naidoo N, Chatterji S: From depressive symptoms to depressive disorders: The relevance of thresholds. British Journal of Psychiatry 2010;196:365–371. 10.1192/bjp.bp.109.071191 20435961

[4] Stanners MN, Barton C a, Shakib S, Winefield HR: Depression diagnosis and treatment amongst multimorbid patients: a thematic analysis. BMC family practice 2014;15:124 10.1186/1471-2296-15-124 24947875 PMC4074384

[5] Fortin M, Hudon C, Haggerty J, Akker M van den, Almirall J: Prevalence estimates of multimorbidity: a comparative study of two sources. BMC health services research 2010;10:111 10.1186/1472-6963-10-111 20459621 PMC2907759

[6] Sutin AR, Terracciano A, Milaneschi Y, An Y, Ferrucci L, Zonderman AB: The trajectory of depressive symptoms across the adult life span. JAMA psychiatry 2013;70:803–811. 10.1001/jamapsychiatry.2013.193 23760442 PMC3740038

[7] Salive ME: Multimorbidity in older adults. Epidemiologic Reviews 2013;35:75–83. 10.1093/epirev/mxs009 23372025

[8] Rast P, Rush J, Piccinin A, Hofer SM: The identification of regions of significance in the effect of multimorbidity on depressive symptoms using longitudinal data: An application of the Johnson-Neyman technique. Gerontology 2014;60:274–281. 10.1159/000358757 24603078 PMC4036629

[9] Aiken LS, West SG: Multiple regression: Testin and interpreting interactions. 1991.

[10] Miller JW, Stromeyer WR, Schwieterman M a.: Extensions of the Johnson-Neyman technique to linear models with curvilinear effects: derivations and analytical tools. Multivariate Behavioral Research 2013;48:267–300. 10.1080/00273171.2013.763567 26741727

[11] Sliwinski MJ, Hofer SM, Hall C, Buschke H, Lipton RB: Modeling memory decline in older adults: the importance of preclinical dementia, attrition, and chronological age. Psychology and aging 2003;18:658–671. 10.1037/0882-7974.18.4.658 14692855

